# Immune Divergences Between the Skin Sensitizer 2,4‐Dinitrochlorobenzene and the Respiratory Sensitizer Phthalic Anhydride Revealed by Mouse Single‐Cell Transcriptomics

**DOI:** 10.1155/jimr/7515115

**Published:** 2026-08-11

**Authors:** Mélanie Mourot-Bousquenaud, Elsa Gratianne Guillot, Amélie Coiscaud, Julianne Mathiot, Samuel Muller, Quentin Fort, Sandrine Jacquenet, Fabrice Battais

**Affiliations:** ^1^ Toxicology and Biomonitoring Division, French Research and Safety Institute for the Prevention of Occupational Accidents and Diseases (INRS), Vandoeuvre-les-Nancy, France; ^2^ Computational Biology Division, INSERM U1052-CNRS UMR5286, Cancer Research Center of Lyon, Leon Berard Center, Claude Bernard Lyon1 University, Lyon, France, centreleonberard.fr; ^3^ Synergie Lyon Cancer Foundation, Lyon, France; ^4^ KidsCaN Team, CNRS UMR5286, INSERM U1052, Cancer Research Center of Lyon, Claude Bernard Lyon 1 University, Lyon, France, univ-lyon1.fr

**Keywords:** dendritic cells, sensitization, single-cell transcriptome, T lymphocytes

## Abstract

**Background:**

Chemical‐induced sensitization is a major health concern. To date, no internationally accepted method is able to discriminate a skin from a respiratory sensitizer. The identification of an immune profile specific to skin or respiratory sensitizers is therefore scientifically relevant.

**Methods:**

Female BALB/c mice were dermally exposed to a skin (2,4‐dinitrochlorobenzene, DNCB) or to a respiratory sensitizer (phthalic anhydride, PA) at day 0 (D0) and D5 to initiate allergic sensitization and at D10, D11, and D12 to induce elicitation. Auricular lymph nodes (LNs) were collected at D0, D3, D7, D10, and D13. Single‐cell transcriptomic and flow cytometric analyses were performed in order to identify immune cell proportions and marker expression. Enrichment analyses were performed at D13 on dendritic and T cells. Cytokine measurements were performed in LN cells’ supernatant by bead array cytometry.

**Results:**

Exposure to both sensitizers induced a strong immune reaction demonstrated by an increase of mature B cells and an evolution of the T cell subpopulations, with an increase of follicular and memory T cells. The proportion of NKT, Th2, and Th9 cells and the levels of IgE increased specifically during PA exposure. Follicular, memory, and helper T cells showed distinct transcriptomic responses to DNCB or PA. The proportion of dendritic cells (DCs) strongly increased starting from D10 in mice exposed to DNCB, and those cells showed a specific transcriptomic signature towards the skin sensitizer. Enrichment analyses suggested a metabolic shift in DCs exposed to DNCB. Single‐cell transcriptomic data were confirmed by flow‐ and bead array cytometry.

**Conclusion:**

A distinct transcriptomic signature was identified in immune cells during sensitization to DNCB or PA in this study.

## 1. Introduction

Repeated contacts with low molecular weight chemicals, also called haptens, can lead to the development of allergic diseases. Allergy affects almost 20% of the European population, and its prevalence is increasing in industrialized countries [1, 2]. The immunological mechanisms leading to the development of allergy occur in two distinct phases: the asymptomatic sensitization phase that initiates allergy and the elicitation phase, which begins after repeated exposures to the chemical and leads to the development of clinical symptoms. The nature of the symptoms observed depends on the immune mechanisms triggered by chemical exposure. Respiratory sensitizers are generally associated with type 2 helper T cells (Th2) –skewed immune responses, characterized by IgE production and eosinophilic inflammation. In contrast, skin sensitizers have classically been linked to type 1 helper T cells (Th1)–dominated responses underlaying delayed‐type hypersensitivity. However, increasing evidence indicates that skin sensitization can elicit more complex and heterogeneous T‐helper responses, depending on the chemical nature, exposure conditions, and microenvironmental factors.

In the context of health prevention, it is necessary to be able to identify sensitizers in order to avoid exposure of employees in the workplace. It should be noted that, unlike skin sensitizers, there is currently no internationally validated test capable of identifying respiratory sensitizers.

The different steps leading to respiratory allergic symptoms are summarized in the form of a proposed toxicological adverse outcome pathway (AOP 39) [3]. In brief, the covalent binding of a hapten to respiratory epithelial nucleophilic proteins initiates the process through key event 1 (KE1). Then, danger‐associated molecular patterns, released under oxidative stress and cell damage, activate epithelial cells through pattern recognition receptors. This leads to the activation of signaling pathways and to the subsequent secretion of proinflammatory cytokines by epithelial cells during KE2. Dendritic cells (DCs) are activated by the haptenated proteins and proinflammatory cytokines and initiate migration to the local lymph nodes (LNs) in KE3. Alternatively, experimental data indicate that the covalent binding of haptens to epithelial proteins can directly activate DCs (from KE1 leading to KE3). During KE4, activated DCs present the sensitizer’s antigen to the naive T cells, which in turn are activated and then start proliferation and differentiation. Following renewed exposure to the same hapten, activated T cells induce the clinical symptoms of allergy. The development of this AOP is part of a project conducted by the International Council on Animal Welfare in OECD programs since 2013 [38]. Nevertheless, several points remain to be clarified to describe the effects of respiratory sensitizers during this process, such as the description of the phenotypic properties of the activated DCs during KE3 or the identification and characterization of the T cell subpopulations activated by respiratory chemical allergens during KE4 [4].

Single‐cell RNA sequencing (scRNA‐seq) is an innovative method through which gene expression profile at the single‐cell level can be achieved. Computer analysis of scRNA‐seq data enables the single‐cell transcriptome to be studied in the context of the cells’ microenvironment. In particular, it can be used to decipher the immune cell transcriptome in sensitized animals [5, 6]. To the best of our knowledge, no study has investigated the transcriptome of DCs and T cells in the LN of chemically sensitized mice.

Understanding the evolution of immune cells and the regulation of transcriptomic markers during chemical‐induced allergic sensitization is essential for implementing new methods for risk assessment. In the present study, we performed LN scRNA‐seq analysis of mice dermally exposed to the reference skin sensitizer 2,4‐dinitrochlorobenzene (DNCB) or to the predominantly respiratory sensitizer phthalic anhydride (PA) in order to identify the immune cell evolution and transcriptomic signature related to these two chemical representatives of the skin and respiratory sensitizer categories. Although PA is a well‐known respiratory sensitizer associated with occupational asthma and IgE‐mediated responses, it is also classified as a skin sensitizer. However, it is important to bear in mind that its ability to cause delayed‐type skin sensitization and allergic contact dermatitis is limited and inconsistent. PA exemplifies a chemical for which the immunological pathways leading to respiratory sensitization are preferentially engaged. This dissociation makes PA a relevant model compound for investigating immune mechanisms and biomarkers specifically associated with the typical Th2‐oriented response induced by respiratory sensitizers [7].

Dermal exposure is a well‐established and controlled approach to trigger antigen uptake, DC activation, and T cell priming, allowing direct comparison between two chemical sensitizers. By applying scRNA‐seq to auricular LN, the objective of this study was to capture and compare the divergent immunological programs engaged by DNCB and PA at single‐cell resolution, independent of the target organ of disease.

Overall, this study could contribute to our understanding of the sensitization process by examining the evolution of immune cell composition and transcriptomic changes during exposure to typical skin and respiratory sensitizers.

## 2. Material and Methods

### 2.1. Mouse Model of Allergic Sensitization

**Figure 1:**
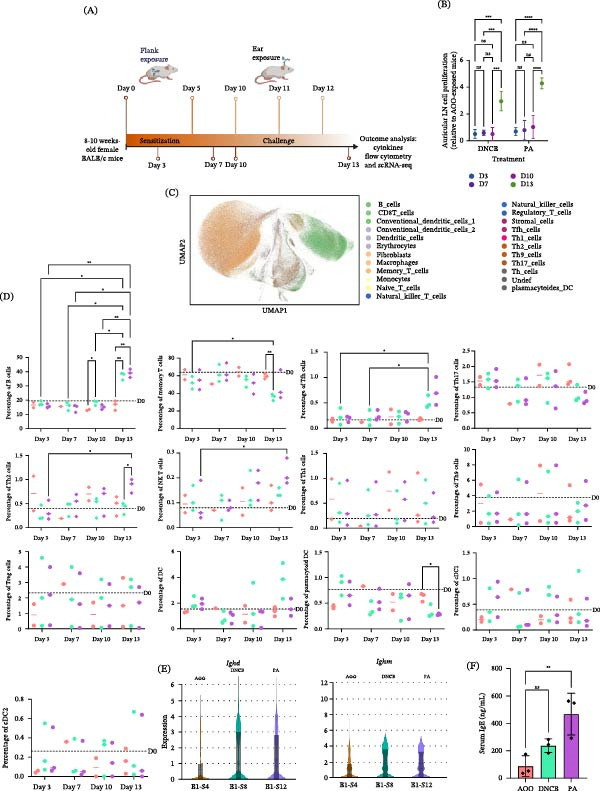
Distinct LN cell composition in mice after exposure to DNCB, PA, or AOO. (A) Experimental study workflow. (B) Stimulation index of viable auricular LN cells at D3, D7, D10, and D13 relative to AOO‐treated mice’ LN cells at the same time points. (C) UMAP plot depicting LN cell composition at D13 in three integrated batches. (D) Percentage of LN cell populations identified with transcriptomic data (orange dots: AOO, green dots: DNCB, and violet dots: PA). (E) Representative violin plots of three independent experiments of *Ighd* and *Ighm* transcripts in LN from mice exposed to AOO, DNCB, or PA. (F) Total serum IgE in mice exposed to AOO, DNCB, or PA. (B, D, F) *n* = 2–3 per treatment. (B, F) Error bar: mean value ± SEM. *p*‐Values were determined by a two‐way (B and D) or one‐way (F) ANOVA followed by a Tukey’s (B and D) or Dunnett’s (F) multiple comparisons test.  ^∗∗^ *p* < 0.01,  ^∗∗∗^ *p* < 0.001, and  ^∗∗∗∗^ *p* < 0.0001.

Female BALB/c mice were purchased from Charles River Laboratories (Saint Germain Nuelles, France). After a 1‐week acclimatization period, 8‐ to 10 week‐old mice were dermally sensitized to the reference respiratory sensitizer PA [8] or to the reference skin sensitizer DNCB [9], as previously described [10]. Control mice were exposed to the vehicle acetone/olive oil (AOO). Briefly, mice were shaved on both flanks 1 day prior to the first exposure. On day 0 (D0) and D5, mice were exposed on each shaved flank (2 ^∗^50 µL) to chemical solutions in order to induce allergic sensitization. On D10, D11, and D12, mice received challenge doses of 2 ^∗^25 µL of the same chemical or vehicle on the dorsum of both ears. Mice were exposed to one sensitizing concentration “*C*” of each chemical (PA 12.5% and DNCB 1%) at D0 and D5 and to one challenging concentration “*C/4*” corresponding to one quarter of the sensitizing concentration (PA 3.12% and DNCB 0.25%) at D10, D11, and D12. The tested concentrations were selected based on preliminary dose‐finding assays to achieve measurable and biologically relevant immunogenic responses for each animal [10]. Using identical nominal concentrations would not necessarily provide a meaningful comparison because of the differences in sensitizing potency between the two typical compounds. The vehicle AOO was always used in a 4:1 (v/v) ratio. Mice were sacrificed at D3, D7, D10, and D13. Three mice were sacrificed without any treatment to represent the D0 condition. Auricular LN and blood were collected each day of sacrifice. Three independent biological replicates, also called batches, were performed for scRNA‐seq analysis. Two additional groups of three to eight mice per condition were used to confirm by flow cytometry and enzyme‐linked immunosorbent assay (ELISA) the evolution of immune cells and the expression of markers observed with transcriptomic data. The experimental procedure is described in Figure 1A. Mice were housed at 22 ± 1°C and 55 ± 5% humidity and had access to standard diet and fresh water ad libitum. Improvement of their well‐being was ensured by nestlets and polycarbonate house addition. Animal experiments were conducted in accordance with the EC Directive 2010/63/EU and with the approval of the local Ethical Committee and the French Ministry for Research and Higher Education (APAFIS# 37817). The animal experiments were conducted in compliance with the ARRIVE guidelines. The animal facility has been approved by the French Ministry of Agriculture (Agreement #D54‐547‐10).

### 2.2. LN Single Cell Preparation for Droplet‐Based scRNA‐Seq and Measurement of Proliferation

Right and left auricular LN were harvested at D0, D3, D7, D10, and D13. After being collected, LN were immediately prepared for droplet‐based scRNA‐seq. Briefly, right and left LN were pooled for each mouse and filtered through a 40 µm filter in cold RPMI 1640 (20 mM Hepes, phenol red) containing 10% fetal calf serum (FCS). After centrifugation, the cells were resuspended in cold phosphate‐buffered saline (PBS) at a cell density of 1000 cells/µL. Cell density and viability were determined automatically (Vi‐CELL, Beckman Coulter).

We determined LN cell proliferation as measured by a stimulation index for each mouse and at each time point, according to the following formula:

Total viable cells in both right and left LN in chemically treated mice ∗ 100Mean of total viable cells in both right and left LN in vehicle treated mice at the same time point/100.

### 2.3. Droplet‐Based scRNA‐Seq

Experiments were performed on the 10x Genomics Chromium platform with the Chromium Next Gem Single Cell 3′ Kit v3.1 and according to the manufacturer’s instructions. Briefly, 20,000 cells resuspended in cold PBS were loaded in each channel. Cells were then partitioned into gel beads in emulsion in the Chromium controller. Afterwards, cell lysis and barcoded reverse transcription of RNA occurred in a thermocycler (Mastercycler nexus, Eppendorf), followed by amplification, DNA fragmentation, Illumina adaptor ligation, and sample dual indexing. Quality control of the libraries was performed with a Fragment Analyzer (Agilent Technologies).

### 2.4. Single‐Cell Sequencing, Alignment and Quality Controls

Barcoded single‐cell libraries were sequenced with 150 bp paired‐end reads on an Illumina NovaSeq 2500 instrument (Azenta Genewiz, Germany). The quality of the reads obtained was then assessed using FastQC software [11]. Cross‐species contamination was checked with Fastq Screen software [12]. Reads were trimmed to remove adapter sequences and were aligned on the mouse genome GRCm39 using CellRanger v7.1.0 (10x Genomics), mapped to one gene, and assigned to one cell. Quality control and filtering of damaged and dead cells were performed based on four metrics: (1) Multiple sequencing of the same molecule (identified by its unique molecular identifier or UMI) was eliminated; 2) knee plots were generated based on UMI counts vs. cells; (3) scatter plots were generated based on the number of detected genes vs UMI per cell to exclude doublets; and 4) the distribution of detected genes per cell. Cells with less than 3500 UMI were filtered. Genes expressed in less than 1% of the cells were excluded. Given the intrinsic differences in mitochondrial content between DCs and T cells, mitochondrial gene percentage was not used as a strict global filtering criterion. Instead, cells with extreme mitochondrial gene values (>35%) and low gene detection were excluded prior to cell type annotation, and mitochondrial content was subsequently evaluated within each cell population. Finally, the number of unique reads, genes, and cells obtained after these steps was analyzed. Sample normalization was performed based on total read counts per cell.

### 2.5. scRNA‐Seq Data Analysis

#### 2.5.1. Cell Population and Cell Density Analysis

Cell types were inferred on batches 1, 2, and 3, using first an automatic method: SingleCellNET [13], then manually annotated using known markers. The list of genes used for each cell type and to perform this annotation is provided in Table S1. A uniform manifold approximation and projection (UMAP) was created and allowed the visualization of major cell types for each batch.

#### 2.5.2. Differential Expression (DE) Analysis

DE tests were performed between adjacent conditions to understand differences across time for each treatment and between treatments for each time point. A *t*‐test was used, and genes were selected based on a corrected *p*‐value (Benjamini–Hochberg correction) of 0.01 and a minimum fold change of 1.5. Tests were performed independently for each batch and for each cell type. Changes in transcriptional cell states were reflected by the number of differentially expressed genes (DEGs).

#### 2.5.3. Integration of Independent Results

The three batches were analyzed independently, as described above; then, the results were integrated using statistical methods, and the process workflow is provided in Figure S1. This method was employed to find DEGs between the two treatments, using the three batches’ data. For each batch, a *t*‐test was performed as described above, and then Fisher’s method [14, 15] was used to integrate the results. On volcano plots, the integrated *p*‐value (from Fisher’s test) is represented versus the mean of the log fold change for the triplicates. The threshold values for DEGs are *p*‐values less than 0.05 and mean log fold changes more than 1.1.

#### 2.5.4. Enrichment Analysis

The sets of DEGs at D13 were annotated with Gene Ontology (GO) database by using R package enrichR (v3.4).

### 2.6. Flow Cytometric Analysis

Two groups of mice were dedicated to flow cytometric analyses. Three to eight mice per condition were exposed as described above to either DNCB, PA, or AOO. The right and left auricular LN were collected at D0, D3, D7, D10, and D13. Immediately after being harvested, both LNs were pooled for each mouse and filtered through a 40 µm filter in cold RPMI 1640 (20 mM Hepes, phenol red) containing 10% FCS. After centrifugation, cells were resuspended in cold PBS‐2 % FCS at a cell density of 4.10^6^ cells/mL. Cells were then blocked with anti‐CD16/32 and stained with antibodies displayed in Table S2 (all from BD Biosciences, France). Cells were analyzed using LSR Fortessa X‐20 and Symphony A1 (Becton, Dickinson and Company), and data were processed with FlowJo software (Becton, Dickinson and Company). The gating strategy for all cell types is provided in Figure S2 and Table S2.

### 2.7. Cytometric Bead Array (CBA) Analysis

At D13, auricular LN cells from animals treated with DNCB, PA, or AOO were cultured with concanavalin A (ConA, 3 µg/mL) for 24 h at a cell density of 5.10^5^ per well. Cells’ supernatants from three mice per condition were collected and stored at −80°C until analysis by CBA. Mouse interleukin (IL)‐2, IL‐4, IL‐5, tumor necrosis factor (TNF), and interferon‐gamma (IFN‐γ) Flex Set (BD Biosciences, France) were used to quantify the levels of Th1/Th2 cytokines secreted into the cell supernatants and according to the manufacturer’s guidelines. The data were acquired on FACSCanto II (Becton, Dickinson and Company) and were analyzed using FCAP Array v3.0 software (Becton, Dickinson and Company).

### 2.8. ELISA

At D13, serum was separated from the whole blood after a 1‐h coagulation step at room temperature by centrifugation (3000 rpm, 20 min, 4°C), and it was then stored at −80°C until analyses. Total IgE levels were measured with the IgE ELISA kit mouse from Invitrogen (France), and CXCL16 levels were measured with the Mouse CXCL16 DuoSet ELISA kit from R&D (France) and according to the manufacturer’s instructions.

### 2.9. Statistical Analysis

Numerical data other than scRNA‐seq data were analyzed using GraphPad Prism 6 (GraphPad Software Inc., USA). For cytometric analyses, results are displayed as % cells and presented as mean ± S.E.M. For ELISA data, results are presented as mean ± S.E.M. For each cell type or cytokine, one‐way ANOVA was applied. When the result was significant, a multiple‐comparison post hoc test (Dunnett or Tukey) was applied to test the difference between the conditions. A logarithmic transformation was applied to the data when residual normality and homoscedasticity conditions were not met. The statistical significance threshold was set at 5%.

### 2.10. Data Availability Statement

Raw Fastq files and processed data provided as AnnData (.h5ad) files have been deposited in the Gene Expression Omnibus database (www.ncbi.nlm.nih.gov/geo) under accession number GSE325403.

### 2.11. Data Limitations

This study did not aim to establish category‐specific characteristics of skin vs. respiratory sensitizers but rather to compare the immune responses induced by two representative compounds of each category, namely, DNCB as a skin sensitizer and PA as a respiratory sensitizer. The generalization applicable to both categories would need additional compound testing in each category.

## 3. Results

### 3.1. A Strong Immune Reaction Was Induced in Mouse LN During Exposure to DNCB and PA

During exposure to the chemicals, cell proliferation was observed in auricular LN and reached at D13 a 2.9‐ and a 4.3‐fold increase in DNCB‐ and PA‐exposed mice, respectively (Figure 1B). Single‐cell analysis of the LN cells was performed in order to identify the immune cell subtype evolution during the procedure. Figure 1C shows the homogenous repartition of the immune cells between the three biological replicates. The cell types were clearly distinct, particularly B and T cells, and the general situation appeared to be the same between the three batches. Additionally, and in order to simplify visualization, 23 UMAPs were generated, one for each cell type, as well as an interactive UMAP (Figure S3A). More precisely, the trend of the evolution of each cell type, depending on the condition, is illustrated in Figure 1D. The three batches had very similar results, which showed the robustness of the single‐cell downstream analyses. There was a strong dynamic for each cell type, with an evolution of cell count through time (Table S3). B cells increased suddenly at D13 and displayed a maturation state in mice exposed to both sensitizers (Figure 1D,E). Serum measurement of total IgE levels showed an increase in PA‐exposed mice (Figure 1F). Regarding T cells, memory T cells (Tmem) first increased with both sensitizers, from D3 to D7, and then decreased (Figure 1D). There was also an evolution of the T helper subpopulation proportions. Both DNCB and PA induced an increase of follicular helper T cells (Tfh) and a decrease of Th17 cells at D13. Furthermore, the proportion of Th2 and natural killer T cells (NKT) increased at D13, only with PA. The proportion of Th1 cells seemed to increase at D13 in the DNCB condition. In addition, the proportion of type 9 helper T cells (Th9) seemed to increase specifically during PA exposure. Regulatory T cells (Treg) remained stable with PA, and they increased between D10 and D13 with DNCB and were overall higher with DNCB than with PA starting from D7. Regarding DCs, they first decrease with both sensitizers and then increase between D10 and D13 only with DNCB. The number of DCs was stable with AOO, confirming a lack of DC‐driven immune reaction to the control. From D3 to D7, the proportion of plasmacytoid DCs (pDCs) decreased in DNCB, while it remained stable with PA. Then, from D10 to D13, they decreased with both sensitizers. The number of type 1 conventional DC (cDC1) and type 2 conventional DC (cDC2) was very low, though the tendency was an increase of cDC1 in mice exposed to DNCB starting from D10. Cytometric analyses confirmed those results, with an increase of B cells at D10, an increase of Tfh cells as soon as D3, an increase of DCs proportion that was higher with DNCB, a higher proportion of Treg at D13 with DNCB, and more activated Th cells at D3 and D13 with both sensitizers (Figure S3B).

### 3.2. DCs Showed a Unique Transcriptomic Signature Towards DNCB

**Figure 2:**
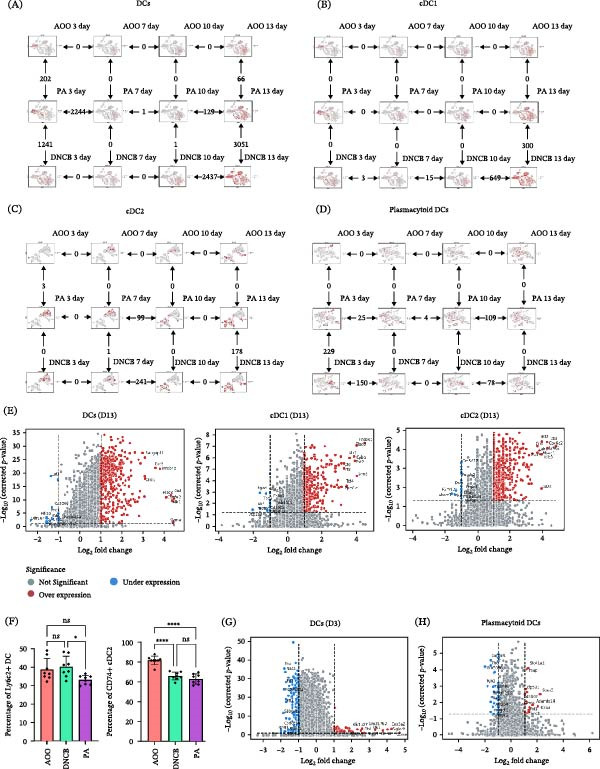
scRNA‐seq identifies a distinct genetic signature in DC subpopulations at D13 in mice exposed to DNCB, PA, or AOO. (A–D) Dendritic cells (DCs), type 1 conventional DCs (cDC1), type 2 conventional DCs (cDC2), and plasmacytoid DCs UMAP plot evolution during time and according to mice treatment. Each data point represents a cell. Numbers indicate DEGs between adjacent conditions. (E) Volcano plots showing the transcriptional effect of DNCB compared to PA condition, in DCs, cDC1, and cDC2 at D13. Each data point in the scatter plot represents a gene. Each gene’s log2 fold change is represented on the *x*‐axis, and the log10 of its *p*‐value is on the *y*‐axis. Genes with a *p*‐value less than 0.05 and a log2 fold change greater than 1.1 are indicated by red dots (representing upregulation). Genes with a *p*‐value less than 0.05 and a log2 fold change less than −1.1 are indicated by blue dots (representing downregulation). (F) Flow cytometric analysis of the percentage of Ly6c2^+^ DCs and CD74^+^ cDC2 in LN from mice exposed to AOO, DNCB, or PA (*n* = 8 per treatment) at D13. (G) DC volcano plot at D3. (H) plasmacytoid DCs volcano plot at D3 scRNA‐seq data: *n* = 3 per condition. Error bar: mean value ± S.E.M. *p*‐Values were determined by a one‐way ANOVA followed by a Tukey’s multiple comparisons test.  ^∗^ *p* < 0.05 and  ^∗∗∗∗^ *p* < 0.0001.

The increase in DC number at D13 was supported by changes in their transcriptional state with both sensitizers (Figure 2A). In this figure, each UMAP represented a condition with its cell density after integration of the three batches. The DEG analysis was represented with the numbers in the arrows between each adjacent condition. There was already a difference in cell state at D3: 1241 DEGs between DNCB and PA conditions. At D3, 202 and 865 genes were differentially expressed between PA vs. AOO and DNCB vs. AOO (data not shown), respectively. Between D10 and D13, we observed a new shift in transcriptional state for both sensitizers: 129 DEGs with PA and 2437 DEGs with DNCB. At D13, there were 3051 DEGs between the two sensitizers, 66 between PA and AOO, and 2007 between DNCB and AOO (data not shown). These results showed a unique transcriptomic signature of DCs to DNCB and PA, with differences that appeared between D3 and D7 with PA and between D10 and D13 with DNCB. In vehicle‐treated animals, no DEGs were found through time.

For cDC1, whose proportion was higher with DNCB at D13, there was a clear change of transcriptional state through time for DNCB, which reached 649 DEGs between D10 and D13. PA and AOO conditions showed no DEGs at any time. At D13, 300 DEGs were found between PA and DNCB. These results showed a unique transcriptomic signature towards the skin sensitizer in cDC1 at D13 (Figure 2B). For cDC2, the transcriptomic changes appeared between D7 and D10 with both sensitizers, with 99 and 241 DEGs for PA and DNCB, respectively. At D13, 178 DEGs were found between the two sensitizers, while no DEGs were found at any time in the AOO condition (Figure 2C). In parallel to their increased proportion at D3, pDCs showed 229 DEGs at D3 between the two sensitizers, and then no transcriptomic differences were observed between them. No change was observed in AOO‐treated mice (Figure 2D).

Figure 2E shows volcano plots to compare both sensitizer’s effects at D13 for subpopulations of DCs. They displayed the most upregulated (in red) or downregulated (in blue) DEGs in DCs from animals treated with DNCB vs. PA. Several genes were upregulated with DNCB: Rabgap1l, Bst2, Tmsb4x, Crip1, Ctsl, Plac8, Cox6a2, Ly6c2, Klk1, and Gzma in DCs; Rnase6, Plac8, Klk1, Cybb, Bst2, Ctsl, Irf8, Grm8, Tcf4, and Ppm1e in cDC1; and Bst2, Ctsl, Cox6a2, Ly6c2, Grn, Lair1, Plac8, macrophage‐expressed gene 1 (Mpeg1), and Iglc3 in cDC2. Significant data from conditions AOO vs. DNCB and AOO vs. PA are shown in Figure S4. By flow cytometric analyses, we observed a significant decrease of Ly6c2^+^ DCs in the LN of mice treated with PA vs. DNCB (33.1% ± 0.9% vs. 40.2% ± 2%). We observed a decrease of CD74^+^ cDC2 in the LN from mice treated with DNCB vs. AOO (65.8% ± 1.4% vs. 81.6% ± 1.5%) and with PA vs. AOO (62.8% ± 1.8% vs. 81.6% ± 1.5%), and the number of CD74^+^ cDC2 was slightly higher in DNCB (Figure 2F).

At D3 in DCs, transcriptomic differences were found between PA and DNCB treatments, although the effects were less pronounced than those observed at D13. The ten most upregulated genes in DCs were Gzma, Siglec‐h, Cox6a2, Grm8, Klk1, Gm21762, Nrxn1, Ctsl, Klk1b27, and Rb3s3. The ten most downregulated genes were the following: Fau, Naca, Eif1, Hsp90ab1, Ftl1, S100a4, Ccdc85a, Ighg1, Ube2c, and Clec4a2. No DEGs were found in cDC1 or cDC2 cells (Figure 2G). In pDCs, transcriptional differences were observed at D3. The ten most upregulated genes were Spns3, Klra3, Adamts14, Prf1, Gzma, Tanc2, Cd300lf, Zfp521, Psap, and Slc41a2. The ten most downregulated genes were Gm5086, Fyb2, Tfrc, Rai2, Tmem163, Carmil1, Inbp4b, Olfr164, Hmgcll1, and P2ry12 (Figure 2H).

GO analysis on DEGs at D13 identified several enriched molecular processes in DCs from the DNCB vs. PA condition. An enrichment of type II interferon response was observed, with three significant GO terms over‐represented: response to type II interferon (9/80 gene counts), regulation of type II interferon production (7/87 gene counts), and cellular response to type II interferon (6/66 enriched gene counts). An enrichment of cell migration/chemotaxis was also seen with three GO terms significantly over‐represented in this process: regulation of cell migration (16/434 gene counts), DC chemotaxis (4/15 gene counts), and DC migration (4/18 gene counts). Under‐represented GO terms were mainly related to protein modification/phosphorylation in DCs with five significant GO terms: protein modification process (175/711 gene counts), peptidyl‐serine phosphorylation (54/158 gene counts), phosphorylation (109/429 gene counts), protein phosphorylation (122/500 gene counts), and peptidyl‐serine modification (53/166 gene counts). In cDC1, only endoplasmic reticulum to Golgi vesicle–mediated transport was under‐represented (8/115 gene counts). In cDC2, a strong positive regulation of genes related to mitochondrial function was observed, with 11 GO terms over‐represented in these cells: NADH dehydrogenase complex assembly (5/53 gene counts), proton motive force–driven mitochondrial ATP synthesis (5/53 gene counts), mitochondrial respiratory chain complex I assembly (5/53 gene counts), aerobic respiration (5/59 gene counts), proton motive force–driven ATP synthesis (5/60 gene counts), oxidative phosphorylation (5/63 gene counts), cellular respiration (5/85 gene counts), mitochondrial respiratory chain complex assembly (5/90 gene counts), aerobic electron transport chain (4/68 gene counts), protein insertion into mitochondrial membrane (3/28 gene counts), and mitochondrial ATP synthesis coupled electron transport (4/70 gene counts) (Figure S5).

### 3.3. T Cells Showed Distinct Responses to DNCB or PA

**Figure 3:**
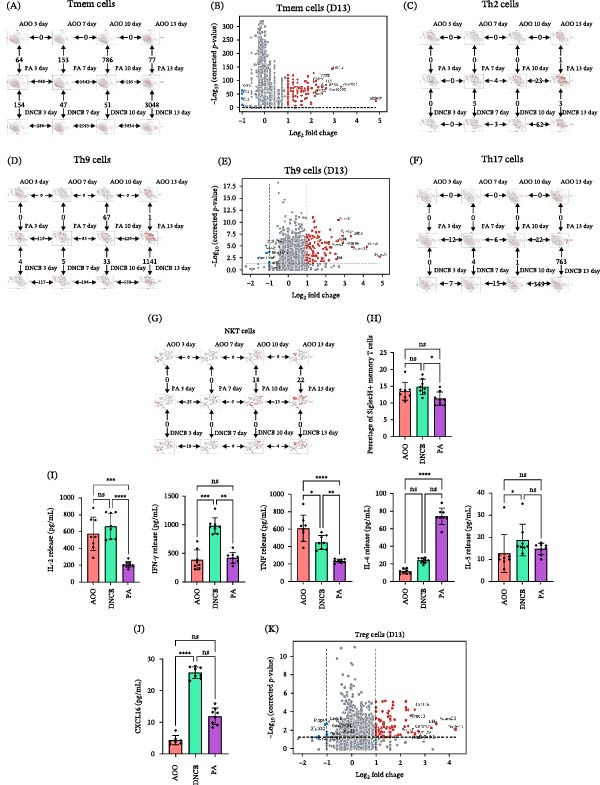
scRNA‐seq identifies a distinct genetic signature in T cell subpopulations at D13 in mice exposed to DNCB, PA, or AOO. (A) Memory T cells (Tmem), (C) type 2 helper T cells (Th2), (D) type 9 helper T cells (Th9), (F) type 17 helper T cells (Th17), and (G) natural killer T cell (NKT) UMAP plots evolution during time and according to mice treatment. Each data point represents a cell. Numbers indicate DE genes between adjacent conditions. Volcano plots showing the transcriptional effects of DNCB compared to PA, in (B) Tmem, (E) Th9, and (K) regulatory T cells (Treg) at D13. Each data point in the scatter plot represents a gene. Each gene’s log2 fold change is represented on the *x*‐axis, and the log10 of its *p*‐value is on the *y*‐axis. Genes with a *p*‐value less than 0.05 and a log2 fold change greater than 1.1 are indicated by red dots (representing upregulation). Genes with a *p*‐value less than 0.05 and a log2 fold change less than –1.1 are indicated by blue dots (representing downregulation). (H) Flow cytometric analysis of the percentage of SiglecH^+^ Tmem in the LN from mice exposed to AOO, DNCB, or PA at D13. (I) Th1/Th2 cytokine release in the supernatant from LN cells of mice exposed to AOO, DNCB, or PA (*n* = 8 per treatment). (J) CXCL16 release from the supernatant from LN cells of mice exposed to AOO, DNCB, or PA (*n* = 8 per treatment). Error bar: mean value ± SEM. *p*‐Values were determined by a one‐way ANOVA followed by a Tukey’s (H, I) multiple comparisons test or Kruskal–Wallis followed by a Dunn’s multiple comparisons test (I, J).  ^∗^ *p* < 0.05,  ^∗∗^ *p* < 0.01,  ^∗∗∗^ *p* < 0.001, and  ^∗∗∗∗^ *p* < 0.0001.

In Tmem cells (Figure 3A), there was a change in transcriptomic state through time in both conditions; this change was the strongest at D13: 3048 DEGs between DNCB and PA, 2126 DEGs between AOO and DNCB (data not shown), and 77 DEGs between AOO and PA. AOO‐treated animals showed constant Tmem transcriptomic state. No DEGs were identified in Th1 cells that were specifically expressed between the two sensitizers’ treatments (data not shown). Th2 cells’ transcriptomic state evolved with both sensitizer treatment, and we found five DEGs in those cells at D7: AY036118 (which is a long noncoding RNA), Camk1d, Cdk8, Cmss1, and Gm42418 and three DEGs at D13: Gata3, Hax1, and Hivep2 (Figure 3C). No effect was observed in AOO‐treated animals. In Th9 and Th17 cells, the number of DEGs increased through time, with 1141 and 763 DEGs, respectively, found at D13 between DNCB and PA. No effect on cell state was identified in AOO treatment (Figure 3D,F).

In Th9 cells, several genes were upregulated at D13: Eldr, Mpeg1, Cybb, Tyrobp, Fmnl2, Rnase6, Adam23, Zeb2, Grm8, and Siglec‐h. Stap2, Ifit1, Fam135a, Fam131a, Mab21l3, and Zc2hc1a were the most downregulated genes in these cells at D13 (Figure 3E). NKT cells changed their state between D3 and D7 with both sensitizers, but no DEG was found between DNCB and PA at any time (Figure 3G).

The effect of DNCB or PA on T cells’ transcriptomic state was strong in Tmem cells at D13, as shown with very low corrected *p*‐values on the associated volcano plot (Figure 3B). In those cells, several genes were upregulated with DNCB: Siglec‐h, Adam23, Fmnl2, Tyrobp, IL‐15, Cadm1, Ankrd33b, Gm16685, Mreg, and Grm8; and downregulated: St13, Ifit1, Ifit3, and Foxp3 (Figure 3B). Siglec‐h, which was the most upregulated gene, was confirmed by flow cytometry (Figure 3H). T cell volcano plots from animals treated with DNCB vs. AOO and PA vs. AOO are displayed in Figure S6 (volcano plots with no significantly regulated genes are not displayed).

While B cells strongly increased at D13 with both sensitizers, no gene was found differentially expressed, either between treatments or as a function of time (data not shown).

Supernatants from LN cells cultured for 24 h in the presence of ConA were assayed for production of several cytokines, representative of the Th1/Th2 balance. LN cells derived from mice exposed to DNCB produced significantly higher amounts of IL‐2, INF‐γ, and TNF, as compared to LN cells coming from animals exposed to PA. Interestingly, cells from mice exposed to PA produced larger quantities of IL‐4, as compared to DNCB‐exposed mice cell cultures. No difference in IL‐5 release was observed (Figure 3I). We found CXCL16 secretion significantly increased in LN cells from DNCB‐treated mice, compared to AOO‐treated mice: 25.7 ± 0.6 vs. 4.4 ± 0.6 pg/mL. It was not the case in PA‐treated mice: 11.9 ± 0.9 vs. 4.4 ± 0.6 pg/mL (Figure 3J). These results were in agreement with the increase of CXCL16 transcript in Treg with DNCB (Figure 3K).

GO analysis on DEGs at D13 identified five enriched molecular processes in Tmem cells related to protein phosphorylation in DNCB vs. PA condition: phosphorylation (143/429 gene counts), protein modification process (203/711 gene counts), protein phosphorylation (152/500 gene counts), peptidyl‐serine phosphorylation (67/158 gene counts), and peptidyl‐serine modification (69/166 gene counts). Enrichment related to GTPase activity, DNA transcription, and vesicle‐mediated transport was also seen. In Tmem, under‐representation of GO terms related to viral processes was seen: regulation of viral genome replication (6/67 gene counts), negative regulation of viral process (5/61 gene counts), negative regulation of viral genome replication (4/53 gene counts), defence response to virus (6/189 gene counts), and positive regulation of viral genome replication (3/29 gene counts). This was concomitant with under‐representation of positive regulation of type I interferon production (4/64 gene counts). In Treg, enrichment was observed in the protein phosphorylation process, vesicle‐mediated transport, tryptophan metabolic process, and Fc receptor signaling pathway. In Treg, under‐representation of GO terms related to RNA splicing was seen: RNA splicing, via transesterification reactions with bulged adenosine as nucleophile (15/180 gene counts), mRNA splicing, via spliceosome (16/211 gene counts), and mRNA processing (15/214 gene counts). Mitochondrial respiration was under‐represented with seven significant GO terms: mitochondrial ATP synthesis coupled electron transport (7/70 gene counts), mitochondrial electron transport, NADH to ubiquinone (5/34 gene counts), aerobic electron transport chain (6/68 gene counts), NADH dehydrogenase complex assembly (5/53 gene counts), proton motive force–driven mitochondrial ATP synthesis (5/53 gene counts), mitochondrial respiratory chain complex I assembly (5/53 gene counts), and cellular respiration (6/85 gene counts). In Treg, we also observed a negative regulation of genes related to apoptosis: negative regulation of programmed cell death (17/381 gene counts), negative regulation of apoptotic process (18/482 gene counts), and regulation of apoptotic process (21/705 gene counts). In Th9 cells, enrichment was seen on Fc receptor signaling: Fc receptor mediated stimulatory signaling pathway (11/22 gene counts), Fc‐gamma receptor signaling pathway (11/24 gene counts), and Fc‐gamma receptor signaling pathway involved in phagocytosis (10/20 gene counts). Regulation of intracellular signal transduction was also enriched (34/297 gene counts). Under‐representation of positive regulation of IL‐12 production was observed (4/33 gene counts) (Figure S7).

## 4. Discussion

In this study, mice were dermally exposed to either a reference skin sensitizer (DNCB) or a respiratory sensitizer (PA), and single cell transcriptomic analyses were conducted to investigate differences in the immune responses triggered by these two chemicals. DNCB and PA were used at different concentrations that were described as inducing equivalent immunogenicity in a modified LLNA assay [10], which formed the basis of the experimental procedure used in this study.

Firstly, a strong immune reaction was observed with both sensitizers. During exposure, the number of *Ighd+ Igdm+* naïve B cells increased in the auricular LN from DNCB‐ and PA‐exposed mice, suggesting their recruitment from the bone marrow and progression towards maturation. Total IgE levels were elevated in serum from mice exposed to PA, suggesting class switching to IgE and generation of IgE‐producing plasma cells, which are hallmark characteristics of a Th2 response. More Th2 cells were found in the LN from mice exposed to PA, as well as an increase of IL‐4 and total serum IgE. In mice exposed to DNCB, an increase of IFN‐γ was measured, as well as an increase of Th1 cells. These results revealed that LN cells derived from mice exposed to either DNCB or PA exhibited a Th1 and Th2 profiles, respectively. It was shown that DNCB and PA induced a preferentially Th1 or Th2 profile, respectively, by measuring IFN‐γ and IL‐4 in murine LN [16]. The present study demonstrated that dermal exposure to an anhydride compound such as PA can elicit a Th2‐type immune response in vivo. These findings align with earlier research indicating that repeated topical application of trimellitic anhydride induces a predominantly Th2‐oriented response in mice [37, 17]. While a systematic review of animal models assessing respiratory effects following skin exposure concluded that the evidence for PA was unconvincing [18], our study confirmed that dermal exposure to PA could trigger allergic sensitization characterized by Th2 polarization, although respiratory effects have not been addressed.

Secondly, considering the cellular landscape, the increase in the proportions of Th2, Th9, and Tfh cells in the LN of mice exposed to PA could be indicative of a developing eosinophilic asthma [19]. While Th2 cells are usually described in respiratory sensitization, Th9 cells are a less frequently studied subset of CD4^+^ T cells that secrete IL‐9 and can be complementary to the function of Th2 cells during allergic diseases [20, 21]. To our knowledge, this study is the first one to report the identification of Th9 as a potential feature of the immune response induced by a respiratory sensitizer.

Regarding the transcriptional response of Th cells, few DEGs were found in Th1 and Th2 cells between DNCB and PA, despite their different proportions at D13. While transcriptomic changes indeed occurred between D7 and D13 in Th2 cells, only five and three DEGs were identified at those time points. No DEGs were found in Th1 cells. One hypothesis is that protein regulation could have occurred and prevented detectable transcriptional differences despite functional differences observed in Th1 and Th2 cells. Cytokine secretion (e.g., IFN‐γ for Th1 and IL‐4 for Th2) might be controlled at the protein level rather than at the mRNA level, and feedback loops in cytokine signaling could minimize detectable gene expression differences [22]. The results concerning Th9 cells are particularly compelling. In addition to their increased proportion over time during PA exposure, these cells exhibit significant transcriptional changes.

In mice exposed to DNCB, we observed a marked upregulation of *Siglec-h* transcript within Treg, Tmem, and Th9 cells compared with mice exposed to PA. Although SIGLEC‐H has been traditionally characterized as a murine pDC–associated receptor that modulates innate sensing and adaptive responses [23, 24], emerging evidence supports a broader regulatory role within lymphoid tissues. The preferential induction of *Siglec-h* in these T cell subsets following DNCB exposure likely reflects a compensatory regulatory program activated by strong Th1 hapten–driven inflammation, which contrasts with the comparatively biased Th2‐skewed response induced by PA exposure.

In mice exposed to DNCB, interferon‐stimulated genes such as *Ifit1* and *Ifit3* were downregulated in both Tmem and Th9, indicating a possible suppression of interferon‐driven responses in T cells. Conversely, *Mpeg1* was upregulated with DNCB, suggesting enhanced cytotoxic activity in Th9 cells. Additionally, *Cybb*, a gene involved in the regulation of oxidative stress, showed increased expression in Th9 cells. Furthermore, glutamate signaling appeared to be upregulated in both Tmem and Th9 cells, as indicated by elevated *Grm8* expression. This finding may reflect broader immunomodulatory effects of this sensitizer.

GO enrichment analysis indicated that DNCB exposure induced a broad upregulation of phosphorylation‐dependent signaling processes in Tmem cells, consistent with enhanced intracellular signaling and functional reprogramming compared to PA. In these cells, the under‐representation of GO terms associated with viral processes suggested a relative attenuation of interferon‐driven antiviral programs compared to PA. This likely reflected functional specialization toward hapten‐specific recall responses under DNCB exposure.

Th9 cells exposed to DNCB displayed enrichment of Fc receptor signaling–related gene ontologies, which suggested enhanced integration of antibody‐dependent signaling in these cells.

In PA‐exposed mice, NKT cells increased. Invariant NKT cells represent a distinct subset of T lymphocytes that bridge innate and adaptive immunity. They are thought to contribute to the development of Th2‐skewed allergic responses and are characterized by their ability to rapidly respond to environmental cues through cytokine production and cytotoxic activity. In particular, they have been shown to be potent inducers of Th2 inflammatory response in a mouse model of ovalbumin‐induced asthma [25]. NKT cells can promote Th2 responses by acting in synergy with Th2 cells. They can be targeted by Th2‐associated factors to exacerbate asthma [26].

With regard to DCs, the *Grm8* gene was upregulated in mice exposed to DNCB. Metabotropic glutamate receptors are involved in neuro‐immune and neuro‐endocrine regulations [27, 28]. To the best of our knowledge, the present study is the first one to demonstrate a regulation of *Grm8* on DCs and in the context of allergic sensitization. Moreover, *Grm8* was also upregulated on Tmem cells after DNCB exposure. It has been shown that during DC‐LT interaction, glutamate can be released by DCs and is a highly effective regulator in the initiation of LT‐mediated immune response [29].

Two genes involved in the regulation of oxidative stress were upregulated in DCs from mice exposed to DNCB: *Cox6a2* and *Cybb*, which are both involved in electron transport and reactive oxygen species regulation. The upregulation of genes involved in oxidative stress, especially in the Nrf2‐Keap1 pathway [30], has been observed in bone marrow‐derived DCs exposed to reference skin and respiratory sensitizers [31].

Our observation of increased *Siglec-h* expression in DCs following DNCB exposure suggests that this regulatory pathway may be engaged beyond the pDC compartment during DNCB‐induced immune activation. Loschko et al. showed that antigen delivery to pDCs via SIGLEC‐H reduces CD4^+^ T cell expansion and Th1/Th17 polarization by maintaining low‐level antigen presentation and limiting effective priming under strong immune stimulation [32]. Its upregulation in DCs in the context of DNCB exposure may therefore reflect an adaptive counter‐regulatory mechanism aimed at constraining excessive DC activation during allergic sensitization. To the best of our knowledge, *Siglec-h* expression has been predominantly described in pDC, and its inducible expression in DCs during sensitization has not been extensively documented, suggesting a previously underappreciated aspect of DC functional plasticity.

*Ly6c2* was upregulated on DCs and cDC2 after DNCB exposure, as compared to PA. This could indicate an inflammatory state of those cells, which was stronger at D13. A study has reported the functional role of CD11b^+^Ly6c^+^ inflammatory DCs in influenza A–facilitated house dust mite sensitization [33].

The under‐representation of mitochondrial respiration and ATP synthesis gene ontologies in DCs from mice exposed to DNCB suggested a relative attenuation of oxidative phosphorylation, consistent with a metabolic shift toward glycolysis. The correlation between DC glycolytic metabolism and immunogenic state has been described in the review from Rodriguez‐Coira et al. [34]. In addition, the over‐representation of GO terms related to cell migration in these cells corroborated the findings from Rodriguez‐Coira et al. on immunogenic DCs. The over‐representation of interferon type II–related GO terms was coherent with the Th1‐polarization profile observed under DNCB exposure. In cDC2, oxidative phosphorylation appeared to be the dominant metabolic pathway engaged in these cells.

Using an in vitro alveolar model, a transcriptomic signature was found in respiratory sensitizers (i.e., isophorone diisocyanate and ethylenediamine) that was distinguishable from two nonsensitizers (i.e., chlorobenzene and dimethylformamide) [35]. While the authors found some upregulated genes in DCs exposed to both sensitizers compared to the nonsensitizers, that is, *Sox9*, *Uaca*, *Ccdc88a*, *Fosl1*, and *Kif20b*, they did not compare the effects of respiratory sensitizers with those of skin sensitizers.

Further research on the analysis of the axillary nodes at early time points could shed light on more localized immune responses occurring near the site of exposure. This approach could enhance the detection of genes regulated at early stages, more effectively than analyses conducted in the more distant auricular nodes. Moreover, it may facilitate the identification of genes involved in the early differentiation of the cDC1 and cDC2 subsets during the initiation phase of sensitization.

LN cell composition and cytokine measurements showed that the route of exposure, that is, the skin, seemed to have no significant influence on T cell polarization. Indeed, certain low‐molecular‐weight chemicals can penetrate the skin, leading to systemic sensitization and, eventually, respiratory symptoms upon subsequent exposure [36]. This highlights the need for considering both inhalation and skin contact when assessing the occupational and environmental risks of sensitizer’s exposure.

To conclude, our data further support the notion that the classical Th1/Th2 paradigm does not fully capture the complexity of chemical‐induced immune sensitization. DNCB exposure selectively induced *Siglec-h* expression across multiple T cell subsets and DCs. This pattern suggests that SIGLEC‐H may act as an inducible regulatory checkpoint in response to strong hapten–driven immune activation. Importantly, functional analyses suggest potential metabolic reprogramming of DCs exposed to DNCB, to the detriment of oxidative phosphorylation. At this stage, the gene expression patterns and cellular effects observed may be specific to the compounds tested. Further investigation with additional known sensitizers should be done to determine whether the panel of DEGs remains consistent between several skin and respiratory sensitizers. Overall, this study provides substantial information that is useful for gaining a better understanding of the effects of two reference sensitizers, DNCB and PA.

## Author Contributions

**Mélanie Mourot-Bousquenaud**: conceptualization, validation, methodology, analysis, manuscript writing (original draft preparation, review, and editing), supervision, project administration. **Elsa Gratianne Guillot and Quentin Fort**: scRNA‐seq analysis. **Amélie Coiscaud**, **Samuel Muller, and Julianne Mathiot**: investigation, cytometric analysis. **Sandrine Jacquenet and Fabrice Battais**: manuscript writing (review).

## Funding

No funding was received for this manuscript.

## Conflicts of Interest

The authors declare no conflicts of interest.

## Supporting Information

Additional supporting information can be found online in the Supporting Information section.

## Supporting information


**Supporting Information** Table S1: List of genes used to annotate the immune cell subpopulations with scRNA‐seq data. Table S2: List of antibodies and gating strategy used for flow cytometric analyses. Table S3: Number and percentage of cells for each cell type and for all of the three batches. Figure S1: Process workflow used to perform the bioinformatic analyses. Figure S2: Gating strategy used to analyze B cells, activated LTh cells, LTfh cells, Treg cells, DCs, Siglec‐H^+^ Tmem cells, Ly6c2^+^DCs, and CD74^+^ cDC2. Figure S3: (A) UMAP plot depicting LN cell density distribution for each of the 23 cell types at D13 in the integrated three batches and interactive UMAP plot at D13, depicting LN cell composition for the integrated three batches. (B) Flow cytometric analysis of immune cells at D3, D10, and D13 in auricular LN. Figure S4: DC volcano plots in comparison to AOO condition. Figure S5: Functional enrichment analysis of selected DCs at D13 in DNCB vs. PA condition. Figure S6: T cell volcano plots in comparison to AOO condition. Figure S7: Functional enrichment analysis of selected T cell types at D13 in the DNCB vs. PA condition.

## Data Availability

The data that support the findings of this study are available from the corresponding author upon reasonable request.
