# Supplementary material for: Immune Divergences Between the Skin Sensitizer 2,4‐Dinitrochlorobenzene and the Respiratory Sensitizer Phthalic Anhydride Revealed by Mouse Single‐Cell Transcriptomics
Source: J Immunol Res. 2026 Aug 11;2026:7515115. doi: 10.1155/jimr/7515115 (PMC13462666; doi:10.1155/jimr/7515115)
Supplement: Supplementary file 1 [file JIMR-2026-7515115-s001.zip › Suppl. Figure 1_Mourot-Bousquenaud-final.pdf]

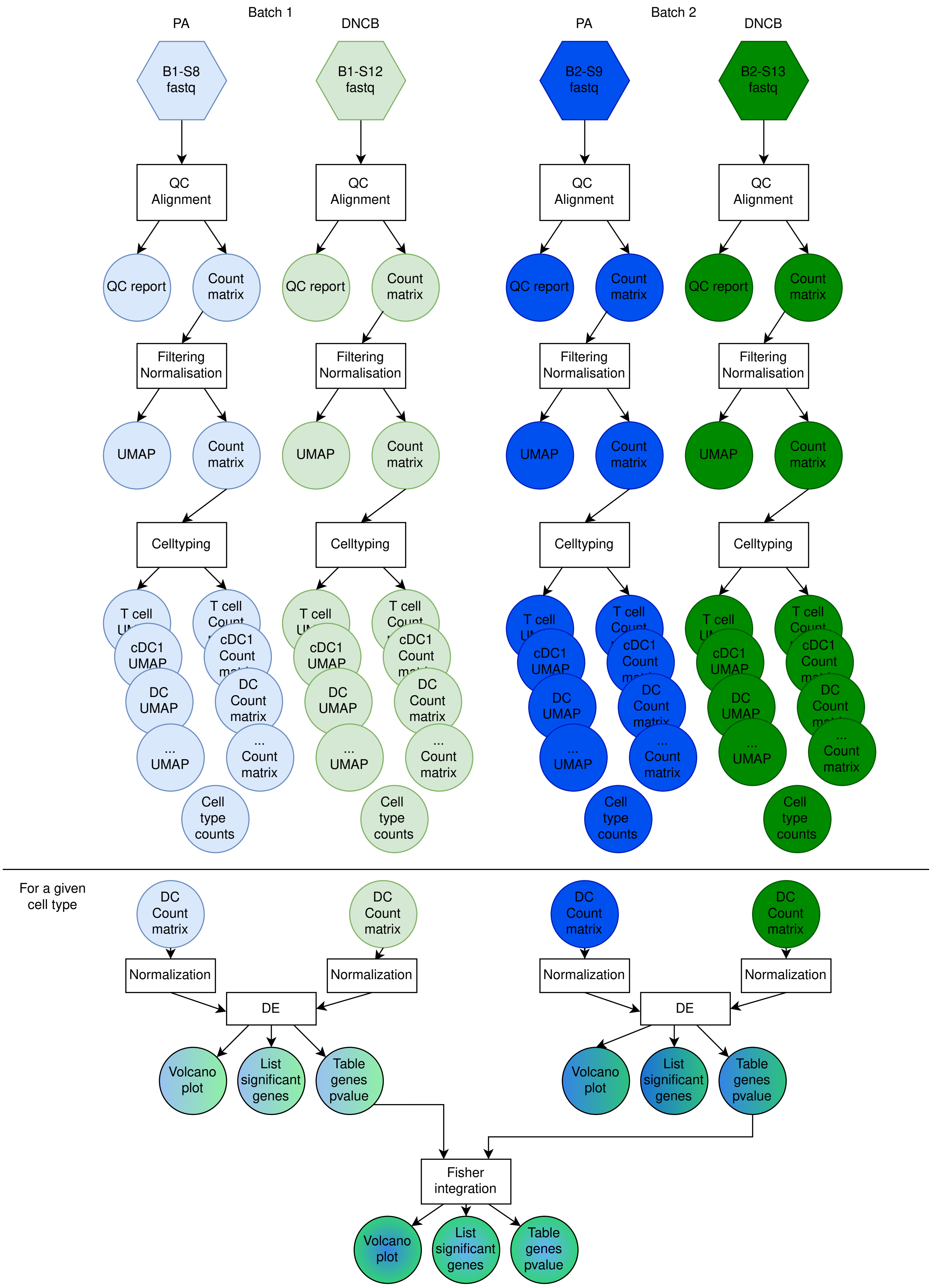

Batch 1
PA
DNCB
Batch 2
PA
DNCB
B1-S8 fastq
B1-S12 fastq
B2-S9 fastq
B2-S13 fastq
QC Alignment
QC report
Count matrix
Filtering Normalisation
UMAP
Count matrix
Celltyping
T cell
cDC1 UMAP
cDC1 Count
DC UMAP
DC Count matrix
... UMAP
... Count matrix
Cell type counts
For a given cell type
DC Count matrix
Normalization
DE
Volcano plot
List significant genes
Table genes pvalue
Fisher integration
Volcano plot
List significant genes
Table genes pvalue
