# Supplementary material for: Immune Divergences Between the Skin Sensitizer 2,4‐Dinitrochlorobenzene and the Respiratory Sensitizer Phthalic Anhydride Revealed by Mouse Single‐Cell Transcriptomics
Source: J Immunol Res. 2026 Aug 11;2026:7515115. doi: 10.1155/jimr/7515115 (PMC13462666; doi:10.1155/jimr/7515115)
Supplement: Supplementary file 1 [file JIMR-2026-7515115-s001.zip › Suppl. Figure 3A_Mourot-Bousquenaud-final.docx]

**(A)**

**(B)**

**Suppl. Figure 3A:** (**A**) UMAP plot depicting LN cell density distribution for each of the 23 cell types at D13 in the integrated three batches (n=3 for each treatment, namely AOO, DNCB and PA). (**B**) Interactive UMAP plot at D13, depicting LN cell composition for the integrated three batches (n=3 per treatment).
