# Supplementary material for: Immune Divergences Between the Skin Sensitizer 2,4‐Dinitrochlorobenzene and the Respiratory Sensitizer Phthalic Anhydride Revealed by Mouse Single‐Cell Transcriptomics
Source: J Immunol Res. 2026 Aug 11;2026:7515115. doi: 10.1155/jimr/7515115 (PMC13462666; doi:10.1155/jimr/7515115)
Supplement: Supplementary file 1 [file JIMR-2026-7515115-s001.zip › Suppl. Figure 3B_Mourot-Bousquenaud-final.docx]

**(B)**

**(C)**

**(A)**

**(E)**

**(D)**

**Suppl. Figure 3B:** Flow cytometric analyses at D3, D10 and D13 of (**A**) B cells, (**B**) activated helper T (LTh) cells, (**C**) follicular helper T (LTfh) cells, (**D**) regulatory T (Treg) cells and (**E**) dendritic cells in LN from mice exposed to AOO (orange dots), DNCB (green dots) or PA (violet dots). Three mice were analysed per group. Error bar: mean value ± SEM. *p-values* were determined by a two-way ANOVA followed by a Tukey’s multiple comparisons test. *p<0.05, **p<0.01, ***p<0.001.
