# Supplementary material for: Immune Divergences Between the Skin Sensitizer 2,4‐Dinitrochlorobenzene and the Respiratory Sensitizer Phthalic Anhydride Revealed by Mouse Single‐Cell Transcriptomics
Source: J Immunol Res. 2026 Aug 11;2026:7515115. doi: 10.1155/jimr/7515115 (PMC13462666; doi:10.1155/jimr/7515115)
Supplement: Supplementary file 1 [file JIMR-2026-7515115-s001.zip › Suppl. Figure 4_Mourot-Bousquenaud-final.docx]

**(A)**

**(B)**

cDC1

DCs

AOO vs DNCB

D13

DCs

**(C)**

**(D)**

Tmem

AOO vs PA

D13

**(G)**

cDC2

**(F)**

cDC1

**(E)**

DCs

**(H)**

Plasmacytoid DCs

AOO vs DNCB

D3

**(I)**

DCs

AOO vs PA

D3

**Suppl. Figure 4:** Volcano plots of (**A**) type 1 conventional dendritic cells (cDC1) and (**B**) dendritic cells (DCs) in AOO vs DNCB conditions at D13, (**C**) DCs and (**D**) memory T cells (Tmem) in AOO vs PA at D13. (**E**) DCs, (**F**) cDC1, (**G**) ) type 2 conventional dendritic cells cDC2 and (**H**) plasmacytoid DCs in AOO vs DNCB at D3. (**I**) DCs in AOO vs PA at D3. The LN from three mice were analysed per condition. Each data point in the scatter plot represents a gene. Each gene’s log2 fold change is represented on the x-axis, and the log10 of its p-value is on the y-axis. Genes with a p-value less than 0.05 and a log2 fold change greater than 1.1 are indicated by red dots (representing up regulation). Genes with a p-value less than 0.05 and a log2 fold change less than -1.1 are indicated by blue dots (representing down regulation).
