# Supplementary material for: Immune Divergences Between the Skin Sensitizer 2,4‐Dinitrochlorobenzene and the Respiratory Sensitizer Phthalic Anhydride Revealed by Mouse Single‐Cell Transcriptomics
Source: J Immunol Res. 2026 Aug 11;2026:7515115. doi: 10.1155/jimr/7515115 (PMC13462666; doi:10.1155/jimr/7515115)
Supplement: Supplementary file 1 [file JIMR-2026-7515115-s001.zip › Suppl. Figure 6_Mourot-Bousquenaud-final.docx]

Tfh

**(C)**

**(B)**

**(A)**

Treg

Tmem

Th9

Th2

Th1

**(E)**

**(D)**

**(F)**

Tfh

**(G)**

**(H)**

Th17

AOO vs PA

D13

🡪

AOO vs DNCB

D13

🡨

Th1

Treg

Tmem

**(J)**

**(I)**

**(K)**

**Suppl. Figure 6:** Volcano plots of (**A**) memory T cells (Tmem), (**B**) follicular helper T cells (Tfh), (**C**) regulatory T cells (Treg), (**D**) type 1 helper T cells (Th1), (**E**) type 2 helper T cells (Th2), (**F**) type 9 helper T cells (Th9) and (**G**) type 17 helper T cells (Th17) in AOO vs DNCB conditions at D13; and of (**H**) Tfh, (**I**) Th1 and (**J**) Treg and (**K**) Tmem in AOO vs PA conditions at D13. The LN from three mice were analysed per condition. Each data point in the scatter plot represents a gene. Each gene’s log2 fold change is represented on the x-axis, and the log10 of its p-value is on the y-axis. Genes with a p-value less than 0.05 and a log2 fold change greater than 1.1 are indicated by red dots (representing up regulation). Genes with a p-value less than 0.05 and a log2 fold change less than -1.1 are indicated by blue dots (representing down regulation).
