# Supplementary material for: Immune Divergences Between the Skin Sensitizer 2,4‐Dinitrochlorobenzene and the Respiratory Sensitizer Phthalic Anhydride Revealed by Mouse Single‐Cell Transcriptomics
Source: J Immunol Res. 2026 Aug 11;2026:7515115. doi: 10.1155/jimr/7515115 (PMC13462666; doi:10.1155/jimr/7515115)
Supplement: Supplementary file 1 [file JIMR-2026-7515115-s001.zip › Suppl. Figure 7_Mourot-Bousquenaud-final.docx]

Over-represented GO terms

Under-represented GO terms

**(A)**

**(B)**

**Suppl. Figure 7:** Functional enrichment analysis of selected T cell types at D13 in DNCB vs PA condition (n=3). (**A**) Dotplot graphs of p value for selected GO terms identified as over-represented in memory T cells (Tmem), regulatory T cells (Treg) and type 9 helper T cells (Th9 cells). (**B**) Dotplot graphs of p value for selected GO terms identified as under-represented in Tmem, Treg and Th9 cells. Significant gene counts for the corresponding pathways are proportional to the circle size.
