# Supplementary material for: Immune Divergences Between the Skin Sensitizer 2,4‐Dinitrochlorobenzene and the Respiratory Sensitizer Phthalic Anhydride Revealed by Mouse Single‐Cell Transcriptomics
Source: J Immunol Res. 2026 Aug 11;2026:7515115. doi: 10.1155/jimr/7515115 (PMC13462666; doi:10.1155/jimr/7515115)
Supplement: Supplementary file 1 [file JIMR-2026-7515115-s001.zip › Suppl. Table 2_Mourot-Bousquenaud-final.docx]

**(A)**

**(B)**

**Suppl. Table 2:** (**A**) List of antibodies used for flow cytometric analyses and their respective isotypes control. (**B**) Gating strategy used to analyse the cell populations of interest in the LN by flow cytometry.
